# Delayed CPAP-Induced Pneumocephalus and Meningitis Posttranssphenoidal Surgery

**DOI:** 10.1155/2021/8855879

**Published:** 2021-08-03

**Authors:** Tara D'Ignazio, Mary Francispillai, Marc Giroux, Martin Albert

**Affiliations:** ^1^Service des Soins Intensifs, Hôpital du Sacré-Coeur de Montréal, Département de Médecine, Université de Montréal, Montréal, Canada; ^2^Département de Chirurgie, Hôpital du Sacré-Coeur de Montréal, Université de Montréal, Montréal, Canada; ^3^Équipe de Recherche en Soins Intensifs (ERESI), Centre de Recherche de l'Hôpital du Sacré-Coeur de Montréal, Université de Montréal, Montréal, Canada

## Abstract

Transsphenoidal surgery (TSS) is a frequently used technique to remove pituitary adenomas. Rare complications of TSS include development of postoperative pneumocephalus. Many patients undergoing TSS also suffer from obstructive sleep apnea (OSA) and thus require positive pressure ventilation. The exact timing of when to safely reintroduce the CPAP machine in this subset of patients is presently not exactly known but is most often cited as being two to four weeks postoperatively. In this case, we describe the story of a 69-year-old female who underwent TSS for a nonsecreting pituitary adenoma in April 2012 and went on to develop pneumocephalus five weeks postoperatively after reintroduction of her CPAP machine. This is the latest presentation of pneumocephalus after reintroduction of CPAP documented in present literature. The case reopens the debate as to how many weeks postoperatively positive pressure ventilation should be withheld to prevent the development of pneumocephalus in patients having undergone TSS with simultaneous OSA.

## 1. Introduction

Transsphenoidal surgery (TSS) is the main approach used for removal of pituitary tumours. [1] Many patients undergoing transsphenoidal surgery have concurrent OSA (obstructive sleep apnea) in part due to the association between Cushing syndrome and acromegaly with OSA. [2] OSA patients tend to complicate more postoperatively, particularly in terms of postoperative desaturation and airway complications. [3] Although the use of CPAP has been demonstrated to decrease such postoperative risks, it is contraindicated in patients having undergone recent transsphenoidal surgery due to the increased incidence of tension pneumocephalus [4].

## 2. Case Presentation

We describe here the case of a 69-year-old female with a past medical history notable for a partially resected nonsecreting pituitary macroadenoma. She had presented 20 years prior with acute onset bitemporal hemianopsia and, upon CT scan, was shown to have a nonsecreting pituitary adenoma in contact with the optic chiasm. She underwent a partial transsphenoidal resection of the adenoma in 1993 as the sphenoidal sinus was described as very narrow, and thus, only 60% of the tumour was resected. However, there was no residual interaction with the optic chiasm. The patient was advised that another surgery may be necessary in the future and left the hospital with no residual symptoms. Her past medical history also includes morbid obesity (BMI 58), obstructive sleep apnea-hypopnea syndrome (OSAHS) treated with CPAP since 2004, insulin-dependent type 2 Diabetes, glaucoma, and asthma.

The patient was readmitted for a subsequent surgery after having developed a recurrence of visual symptoms. The patient underwent a transsphenoidal surgery without complication. No suspicion of CSF leak was described in the operative protocol. Her postoperative period was complicated by a left ophthalmic artery occlusion and an acute subarachnoid hemorrhage, which are both documented complications after transsphenoidal surgery. [5] The patient left the hospital three weeks postoperatively for rehabilitation before returning home.

**Figure 1:**
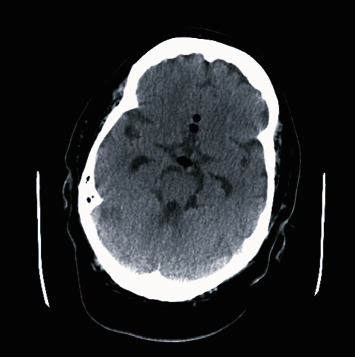
Brain scan demonstrating beginning of pneumocephalus.

**Figure 2:**
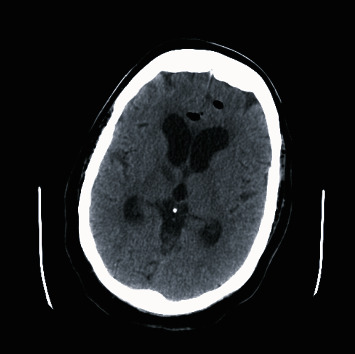
Brain CT demonstrating hydrocephalus and pneumocephalus.

The patient demonstrated good progress during rehabilitation. Nasal CPAP was reintroduced (autoset with a range of 5-12 cm H_2_O) around five weeks postoperatively. Soon thereafter, she rapidly developed fever up to 39C, decreased level of consciousness, and nuchal rigidity, and was subsequently transferred back to our institution. Brain CT scan demonstrated hydrocephalus and pneumocephalus as shown in Figures 1 and 2. Sphenoidoscopy demonstrated left CSF fistula. A lumbar puncture revealed a cerebrospinal fluid with elevated leukocytes, glucose level of 6.8, and lactic acid of 6.8. Cultures did not demonstrate any pathogen. A diagnosis of culture-negative meningitis secondary to reopening of the surgical site by CPAP was postulated. The patient was treated with broad spectrum antibiotics, an external ventricular drain was installed, and the patient was monitored until her discharge.

## 3. Discussion

Presently, positive pressure ventilation such as the use of a CPAP is contraindicated in the immediate postoperative period of TSS due to the increased risk of pneumocephalus. [4] One proposed mechanism for the development of tension pneumocephalus posttranssphenoidal surgery is the ball-valve effect, in which the application of positive pressure through a dural defect allows for the accumulation of air without a way out. [6] In the perioperative period, it is possible to manage hypercapnia and desaturation without use of noninvasive positive pressure ventilation in the majority of cases by using high-flow oxygen face mask in order to avoid such complications. [7] However, the appropriate timing for reintroduction of CPAP posttranssphenoidal surgery has yet to be established.

A recent retrospective study found that out of 64 post-TSS patients with OSA, eight required CPAP in the immediate postoperative period and yet did not develop pneumocephalus. [8] In terms of the reintroduction of CPAP posthospital discharge, another recent retrospective study found that out of 324 patients having undergone transsphenoidal surgery to resect a sellar mass and a total of 349 procedures, the only two patients to develop pneumocephalus did not have OSA or use CPAP. In fact, 69 of their patients did have OSA and were advised that they could resume the use of their CPAP 2-4 weeks postprocedure. None of these patients went on to develop pneumocephalus despite their CPAP use, which vouches for the security of CPAP reintroduction a few weeks after the procedure. [2] Surprisingly, our patient went on to develop pneumocephalus after CPAP introduction 5 weeks postprocedure, which is the latest reported case to date to our knowledge. It is generally accepted that the recovery period after a transsphenoidal procedure can extend up to six weeks postoperatively, with a restriction on heavy weight lifting until twelve weeks postoperatively. [9] This case reopens the debate as to when it is safe to restart CPAP usage posttranssphenoidal procedure. While the installation of a tracheostomy could have been done, the attending physicians at the time considered it a last resort option for such a patient. To impose a tracheostomy procedure for all OSAHS patients after a transsphenoidal surgery would be too invasive but could be considered in patients with a known CSF leak and a high risk of complication without positive pressure ventilation.

## 4. Conclusion

OSA is a frequent problem among patients undergoing transsphenoidal surgery. The immediate use of CPAP posttranssphenoidal surgery is presently contraindicated due to the increased risk of tension pneumocephalus. The appropriate moment to reintroduce CPAP post-TSS, which balances the risks of the development of pneumocephalus with the risks of untreated OSA, remains unknown as pneumocephalus can develop up to five weeks postsurgical intervention as seen in our present case study.

## Data Availability

No data was used to support this study as it is a case report.
